# Synthesis and Properties of Energetic MOFs Based on Bis(3-Nitro-1*H*-1,2,4-triazole-5-yl) Amine: Advancing High Thermal Stability and Low Sensitivity

**DOI:** 10.3390/molecules30122478

**Published:** 2025-06-06

**Authors:** Shiluo Chen, Jinxin Wang, Yuteng Cao, Kangcai Wang, Haijun Yang, Tianlin Liu

**Affiliations:** 1 School of Materials and Chemistry, Southwest University of Science and Technology, Mianyang 621010, China; cslswust@163.com; 2 Institute of Chemical Materials, China Academy of Engineering Physics, Mianyang 621900, China; wangjinxin20@gscaep.ac.cn (J.W.); caoyuteng18@gscaep.ac.cn (Y.C.); wangkangcai@caep.cn (K.W.)

**Keywords:** E-MOFs, alkali metal, BNTA, high thermal stability, low sensitivity

## Abstract

Energetic metal–organic frameworks (E-MOFs) have recently emerged as a promising strategy to address the long-standing challenge of reconciling energy and sensitivity in energetic materials. Nitrogen-rich compounds, with their abundant nitrogen atoms and superior enthalpy of formation, are particularly beneficial for forming multiple coordination bonds while simultaneously elevating the energy content. This makes them ideal ligand molecules for constructing E-MOFs. In this work, we report the synthesis and structural design of a novel series of E-MOFs, constructed from the nitrogen-rich energetic ligand BNTA and a range of alkali metals (Na–Rb, compounds **2**–**5**). The research indicates that the synthesized E-MOFs exhibit high thermal stability and low sensitivity. Specifically, Compound **3** displays a high decomposition temperature of 285 °C, with impact sensitivity and friction sensitivity values exceeding 40 J and 360 N, respectively. Moreover, Compound **3** also exhibits excellent computational detonation performance. Significantly, this study demonstrates how the aromatic character, coordination chemistry, and intermolecular interactions work synergistically to enhance the stability and safety of E-MOFs, thereby establishing fundamental criteria for engineering the next generation of energetic frameworks.

## 1. Introduction

Balancing the inherent contradiction between energy and sensitivity is a crucial challenge in the field of energetic materials [1,2]. Achieving this balance can significantly enhance the comprehensive performance of energetic materials, thereby unlocking their potential for broader applications [3,4,5]. Metal–organic frameworks (MOFs) have emerged as highly promising candidates to address this challenge, owing to their exceptional structural tunability, remarkable chemical and thermal stability, and unparalleled design flexibility [6,7,8]. These attributes have attracted substantial research interest in recent years [9,10,11,12]. The synthesis of MOFs typically involves the straightforward formation of coordination complexes between metallic centers and polytopic organic connectors [13,14]. Energetic MOFs (E-MOFs), a specialized subclass of metal–organic frameworks, represent an emerging category of porous materials formed by integrating high-energy ligands with metal nodes [15]. These materials exhibit remarkable energy density, tunable architectures, and versatile characteristics, making them highly valuable for applications in propellant and explosive technologies [16]. By carefully selecting appropriate ligands, it is possible to construct energetic MOFs (E-MOFs) with diverse interesting structures [17,18,19]. These structures can effectively enhance the thermal stability and safety of the compounds, thereby mitigating the energy-sensitivity trade-off [20,21,22]. This approach not only offers a viable solution to the long-standing dilemma but also paves the way for the development of next-generation energetic materials with optimized performance [23,24].

**Figure 1:**
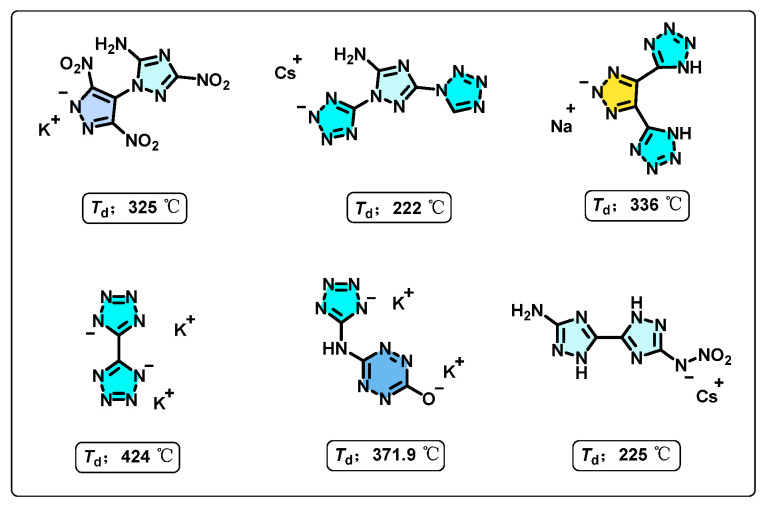
Some reported N-rich E-MOFs with high thermal stability.

It is worth noting that nitrogen-rich (N-rich) ligands, due to their high content of nitrogen and oxygen atoms, can form multiple coordination bonds with metal ions [25,26,27,28]. This leads to the formation of compounds with a high heat of formation (HOF), high density, enhanced thermal stability, good oxygen balance, and environmental friendliness [29,30,31]. As a result, the past decades have witnessed substantial progress in the development of energetic metal–organic frameworks (E-MOFs) employing N-rich coordinating ligands, such as triazoles, tetrazoles, and tetrazines [32,33,34,35]. As illustrated in Figure 1, continuous research efforts have led to the successful synthesis of a series of N-rich E-MOFs that exhibit remarkable thermal stability. E-MOFs incorporating higher nitrogen content and imino-bridged organic ligands tend to display superior thermal stability, as evidenced by their elevated decomposition temperatures [36,37,38,39,40,41]. For instance, potassium [5,5′-bitetrazole]-1,1′-diide (K_2_BT) and potassium 4-(1H-tetrazole-5-ylamino)-1,2,4,5-tetrazine-1-one (K_2_TATZO) exhibit notably high decomposition temperatures of 424 °C and 371.9 °C, respectively. Moreover, the abundance of nitrogen atoms in these ligands allows for extensive metal–ligand coordination, while the ligand–ligand interactions are primarily mediated by weaker forces [42,43,44]. This structural arrangement reduces mechanical sensitivity to external stimuli, thereby enhancing overall safety [45]. The above research findings demonstrate that E-MOFs based on nitrogen-rich energetic ligand molecules hold immense potential as candidates for high thermal stability and low sensitivity explosives [46,47,48].

In this study, BNTA was employed as an organic ligand due to its excellent thermal stability and low sensitivity to external stimuli. The combination with alkali metal cations provides a potential approach to develop E-MOFs with enhanced thermal stability and reduced sensitivity. Accordingly, four novel E-MOFs were constructed from alkali metals and the BNTA^2−^ anion through simple preparation methods. The structures of compounds **2**–**5** were thoroughly characterized, and their physicochemical and energetic parameters were comprehensively evaluated using both experimental and theoretical approaches. These E-MOFs exhibit abundant coordination bonding interactions between the metal centers and energetic ligands, as well as significant hydrogen bonds and π–π interactions among the ligands themselves. These intermolecular interactions collectively contribute to the satisfactory thermal stability and mechanical insensitivity of the compounds. Moreover, compound **3** has the highest performance of energy, thermal stability, and mechanical sensitivity, endowing it with the greatest potential for practical applications.

## 2. Results and Discussion

### 2.1. Single-Crystal Structure

The crystals of compounds **3**–**5**, suitable for X-ray diffraction, were prepared by evaporation in aqueous solution. Compound **3** (CCDC 2454221) crystallizes in the triclinic *P* − 1 space group, with each unit cell containing four asymmetric formula units (Z = 2). Notably, the calculated crystal density of this compound reaches a high value of 1.941 g·cm^−3^ at 295 K.

**Figure 2:**
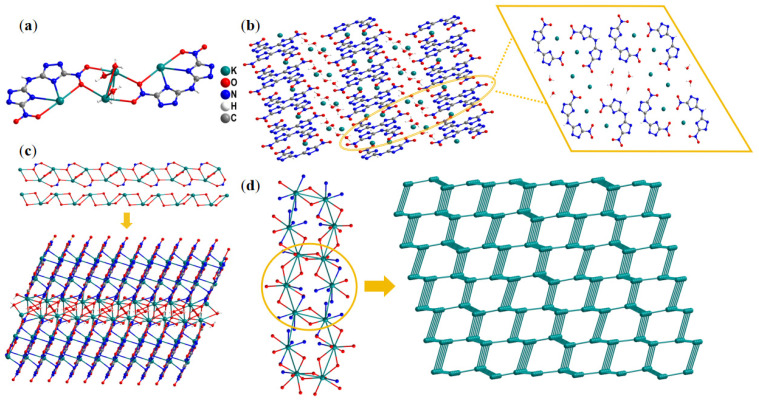
(**a**) Asymmetric unit of compound **3**. (**b**) The layered-like crystal packing of compound **3** and the molecular arrangement in the same layer. (**c**) The chain-like structure and layered structure extended via coordination and hydrogen bonding in compound **3**. (**d**) Simplified unrelated atoms and connected nodes and networks observed along the a-axis.

As shown in Figure 2a, the asymmetric unit comprises four K^+^ (K1, K2, K3, and K4) ions, two BNTA^2−^ anions, and four water molecules. The four K^+^ ions exhibit distinct coordination environments with the surrounding BNTA^2−^ anions and water molecules. Specifically, each K1 and K3 ion is connected to seven neighboring atoms. K1 is coordinated with four nitrogen atoms and three oxygen atoms, whereas K3 is coordinated with three nitrogen atoms and four oxygen atoms. The coordination modes of K2 and K4 are slightly different due to the presence of water molecules in their vicinity. Each K2 and K4 ion are connected to six and seven neighboring atoms, respectively. K2 is coordinated with one nitrogen atom and five oxygen atoms, while K4 is coordinated with one nitrogen atom and six oxygen atoms. The lengths of the K–O coordination bonds range from 2.710 to 3.315 Å, while the K–N coordination bonds span from 2.732 to 3.232 Å.

As a result of these diverse coordination forms, compound **3** exhibits a unique structural arrangement. Specifically, it forms ladder-like layer stackings, as shown in Figure 2b. This arrangement of anions and cations, with the alternating distribution of BNTA^2−^ anions and coordinated K^+^ ions within the same layer, facilitates interlayer sliding and compression. This structural feature effectively reduces mechanical sensitivities toward external stimuli, thereby enhancing the overall stability and safety of the compound.

To further investigate the stacking modes of compound **3**, as shown in Figure 2c, K^+^ ions coordinate with adjacent BNTA^2−^ anions and bridging water molecules via ligand–metal bonds, forming an extended one-dimensional chain-like structure with alternating K–O–K–O connectivity. Meanwhile, BNTA^2−^ anions interact with K^+^ ions through coordination bonds and are regularly distributed around the chain. To observe its three-dimensional structure (Figure 2d), the K^+^ ions in the compound exhibit a hexagonal arrangement when observed from the a-axis direction. To further visually illustrate this phenomenon, by simplifying the N/O atoms around the K^+^ ions, it is found that there is a regular hexagonal coordination geometry between K^+^ ions, forming a honeycomb-like structure.

**Figure 3:**
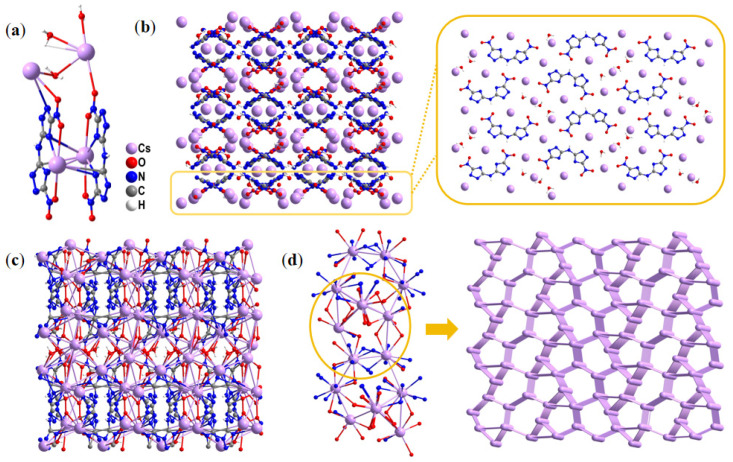
(**a**) Asymmetric unit of compound **4**. (**b**) The layered-like crystal packing of compound **4** and the molecular arrangement in the same layer. (**c**) Complex three-dimensional structure constructed by Cs^+^ ions, BNTA^2−^ anions, and water molecules together. (**d**) Simplified unrelated atoms and connected nodes and networks.

Compound **4** (CCDC 2454222) crystallizes in the *monoclinic P* 2_1_/*c* space group, with each unit cell containing four asymmetric formula units (Z = 4). Remarkably, the calculated crystal density reaches up to 2.731 g·cm^−3^ at 293 K. As shown in Figure 3a, the asymmetric unit contained four Cs^+^ (Cs1, Cs2, Cs3, and Cs4), two DNTA^2−^ anions, and three water molecules. Four Cs^+^ ions have different coordination forms with surrounding BNTA^2−^ anions and water molecules. Each Cs1 and Cs3 is connected to the surrounding BNTA^2−^ anion and water molecules through coordination bonds, interacting with eight neighboring atoms. Cs1 connects to four N atoms and four O atoms, while Cs3 connects to two N atoms and six O atoms. Meanwhile, the number of coordinating atoms of Cs2 and Cs4 is completely different. Cs2 connects to six N atoms and three O atoms, while Cs4 connects to one N atom and five O atoms. These Cs^+^ ions, with distinct coordination modes, are regularly distributed in the holes formed by the alternating and staggered appearance of BNTA^2−^ anions (Figure 3b). The lengths of Cs–O coordination bonds are in the range of 3.101–3.547 Å, yet Cs–N coordination bonds are from 3.102 to 3.760 Å. The staggered arrangement of anions and the regular distribution of cations can effectively release the mechanical sensitivity caused by external stimuli, ensuring the safety of the compound.

To gain insights into the complicated 3D network of compound **4**, we penetratingly dissected the coordination mode of the Cs^+^ ions, BNTA^2−^ ligands, and water molecules. As shown in Figure 3c, Cs^+^ ions construct an intricate 3D network structure through coordination with BNTA^2−^ anions and water molecules. This structural complexity arises from not only the face-to-face staggered arrangement of BNTA^2−^ anions but also the additional perpendicular orientation of some BNTA^2−^ anions relative to the plane formed by these staggered units. To more clearly visualize the 3D arrangement, the non-coordinating atoms in the BNTA^2−^ anions were simplified (Figure 3d). It can be observed that the BNTA^2−^ anions are distributed around the chain-like structures formed by the Cs^+^ ions. When only the Cs^+^ ions are considered, they form polygonal structures of varying shapes. These polygons alternate in arrangement, collectively constructing an intricate 3D network.

**Figure 4:**
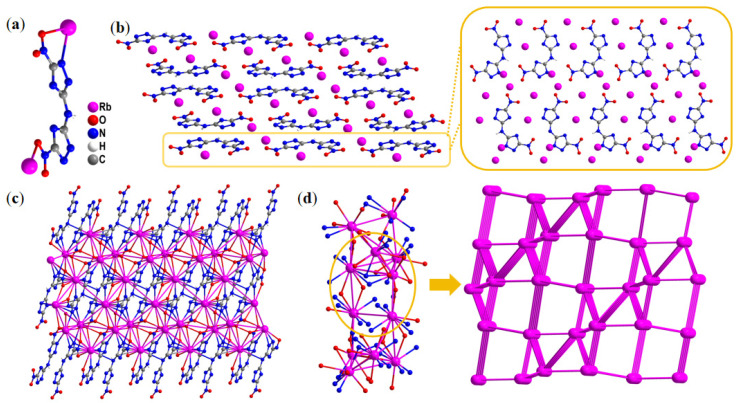
(**a**) Asymmetric unit of compound **5**. (**b**) The layered-like crystal packing of compound **5** and the molecular arrangement in the same layer. (**c**) Complex three-dimensional structure constructed by Rb^+^ ions and BNTA^2−^ anions. (**d**) Simplified unrelated atoms and connected nodes and networks.

Compound **5** (CCDC 2454223) crystallizes in the *monoclinic P* 2_1_/*n* space group, with each unit cell containing four asymmetric formula units (Z = 4). Remarkably, the calculated crystal density reaches up to 2.582 g·cm^−3^ at 150 K. As shown in Figure 4a, the asymmetric unit contained two Rb^+^ (Rb1 and Rb2) and one DNTA^2−^ anion. Two Rb^+^ ions have different coordination forms with surrounding BNTA^2−^ anions. The lengths of the Rb–O coordination bonds are in the range of 2.963–3.417 Å, yet Rb–N coordination bonds are from 2.981 to 3.527 Å. Both Rb1 and Rb2 are connected to the surrounding BNTA^2−^ through coordination bonds, interacting with eight neighboring atoms. Rb1 connects to five N atoms and three O atoms, while Rb2 connects to three N atoms and five O atoms. Compound **5** exhibits the same BNTA^2−^ anion arrangement as compound **3**, but due to the absence of water molecules in its crystal lattice, it displays better layered stacking. Rb^+^ ions are uniformly and regularly distributed around these anions through coordination bonds (Figure 4b). Better-layered stacking, more conducive to intermolecular sliding and compression, endows compounds with better safety.

To further reveal the stacking forms in compound **5**, as shown in Figure 4c, a complex 3D network structure is formed between Rb^+^ ions and planes constructed by misaligned BNTA^2−^ anions. Compared to compound **4**, compound **5** has better intuition and regularity. The single-crystal structure of compound **5** is also simplified to give us insight into the intricate network. In Figure 4d, it can be observed that the Rb^+^ centers are arranged in alternating triangular and quadrilateral configurations, and the structure overall exhibits an irregular arrangement form. Expanded structural illustrations better demonstrate this unique connectivity pattern. Further expansion can better observe this phenomenon.

### 2.2. Thermal Behaviors

Thermal stability is one of the significant indicators for evaluating the safety of energetic compounds, which is related to their potential applications under special conditions. Thermogravimetric analysis (TG) and differential scanning calorimetry (DSC) were applied to investigate the thermal stabilities of compounds **2**–**5** at 5 °C·min^−1^ in the N_2_ atmosphere (Figure 4). Beforehand, compounds **2**–**5** were dried in air.

**Figure 5:**
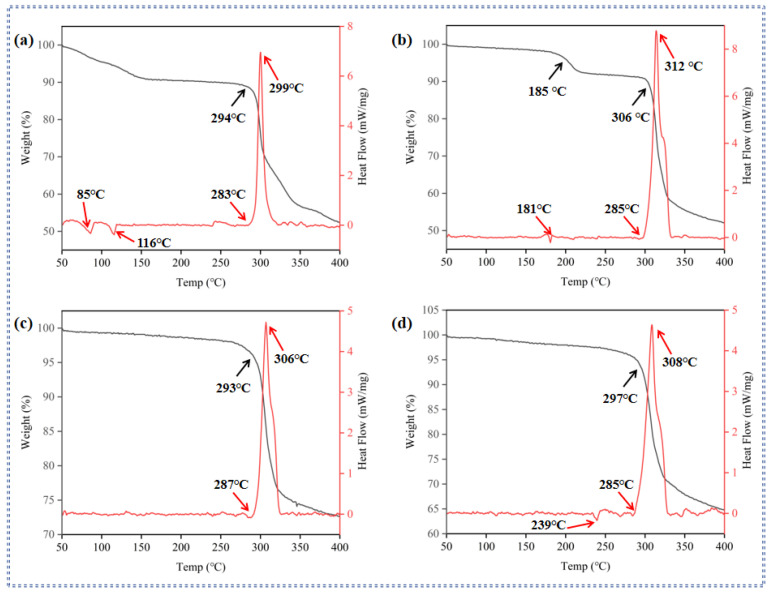
(**a**) TG-DSC curves of compound **2**. (**b**) TG-DSC curves of compound **3**. (**c**) TG-DSC curves of compound **4**. (**d**) TG-DSC curves of compound **5**.

The thermal analysis results demonstrate that all synthesized compounds (**2**–**5**) exhibit excellent thermal stability, with decomposition temperatures ranging from 283 to 287 °C, which is higher than [Cs (ABTNA)H_2_O] _n_ [41]. For compound **2**, the DSC curve (Figure 5a) reveals two endothermic peaks at 85 °C and 116 °C, which correspond to weight loss observed in the TG curve. These peaks are attributed to the volatilization of free water and crystalline water within the compound. Subsequently, a pronounced exothermic peak is observed, with an onset temperature of 283 °C and a peak temperature of 299 °C.

A similar decomposition trend is observed for compound **3** (Figure 5b), which exhibits an endothermic peak at 181 °C, likely due to the evaporation of crystalline water. Compound **3** then displays a remarkably sharp exothermic peak, with an onset temperature of 285 °C and a peak temperature of 312 °C. In contrast, the decomposition curves of compounds **4** and **5** differ significantly from those of compounds **2** and **3**. The TG curves of compounds **4** and **5** each show only one distinct weight-loss step, while their DSC curves exhibit notable differences (Figure 5c,d). For compound **4**, a single sharp and intense exothermic peak is observed in the DSC curve, with an onset temperature of 287 °C and a peak temperature of 306 °C. Compound **5**, on the other hand, displays an endothermic peak at 239 °C without a corresponding mass loss in the TG curve, suggesting that this peak likely represents the melting of compound **5**. This is followed by a sharp exothermic peak with an onset temperature of 285 °C and a peak temperature of 308 °C. Overall, these results indicate that all compounds (**2**–**5**) possess good thermal stability, with high decomposition temperatures and distinct thermal behaviors that reflect their unique structural characteristics.

In addition, the TG-DSC analysis further reveals that compounds **2** and **3** undergo several stages of weight loss and a single stage of heat release, primarily associated with the collapse of the 3D framework and the thermal decomposition of the energetic components [38]. In contrast, compounds **4** and **5** each experience only one stage of weight loss and heat release, also accompanied by the collapse of the 3D framework and the thermal decomposition of the energetic components. From these observations, it can be concluded that the enhanced thermal stability of compounds **2**–**5** is partly attributed to the robustness of their framework structures, which are stabilized by multiple coordination bonds. Generally, a higher number of coordination bonds correlate with increased thermal stability, as these bonds provide additional structural reinforcement and resistance to thermal degradation.

### 2.3. Energetic and Safety Characteristics

Detonation characteristics and mechanical sensitivity are crucial indicators for evaluating the energy output and safety performance of energetic materials. Compounds **2**–**5** exhibited excellent densities, ranging from 1.860 to 2.733 g·cm^−3^, which exceed [Na(H_2_BTT) (H_2_O)_2_] _n_ [38]. The enthalpy of formation (HOF) for these compounds was calculated using the Gaussian 09 software package, while their detonation performance was evaluated using the EXPLO5 (v6.02) code [49], based on the obtained HOF and density values. The enthalpy of formation for compounds **2**–**5** lies between −273.4 and −491.5 kJ·mol^−1^. However, due to the computational limitations of EXPLO5 (v6.02), detonation performance calculations were only performed for compounds **2** and **3**. The results indicated that compound **3** exhibited superior detonation performance, with a detonation velocity of 8844 m·s^−1^ and a detonation pressure of 26.88 GPa. This enhanced performance is primarily attributed to compound 3′s more negative enthalpy of formation (−429.5 kJ·mol^−1^) and higher density (1.941 g·cm^−3^ at room temperature). These calculation results demonstrate that compound **3** is a highly energetic compound with excellent detonation performance, surpassing traditional explosive molecules such as TNT (detonation velocity: 6881 m·s^−1^; detonation pressure: 19.5 GPa) and RDX (detonation velocity: 8750 m·s^−1^) [50,51].

The mechanical sensitivities of the energetic materials were assessed using the BAM standard sensitivity testing method. Prior to testing, compounds **2**–**5** were thoroughly dried to ensure accurate measurements. The experimental results revealed that compounds **2** and **3** exhibited impact sensitivities and friction sensitivities both exceeding 40 J and 360 N, respectively. In contrast, compounds **4** and **5** showed slightly lower sensitivity. Specifically, compound **4** had an impact sensitivity of 15 J and a friction sensitivity of 240 N, while compound **5** exhibited values of 20 J and 288 N, respectively. Despite these minor differences, all four target compounds qualify as low-sensitivity energetic materials, fully meeting the stringent application requirements. Notably, compounds **2** and **3** demonstrated superior mechanical insensitivity, which can be partly attributed to their robust hydrogen-bonding interactions and well-ordered three-dimensional structures. These structural features effectively dampen the mechanical sensitivity induced by external stimuli, thereby enhancing overall safety and stability. Overall, although slight variations in mechanical sensitivity were observed among the four compounds, they all outperformed typical energetic molecules such as RDX [51] and HMX [52] are both in Table 1, highlighting their potential as advanced energetic materials.

### 2.4. Hirshfeld Surface

**Figure 6:**
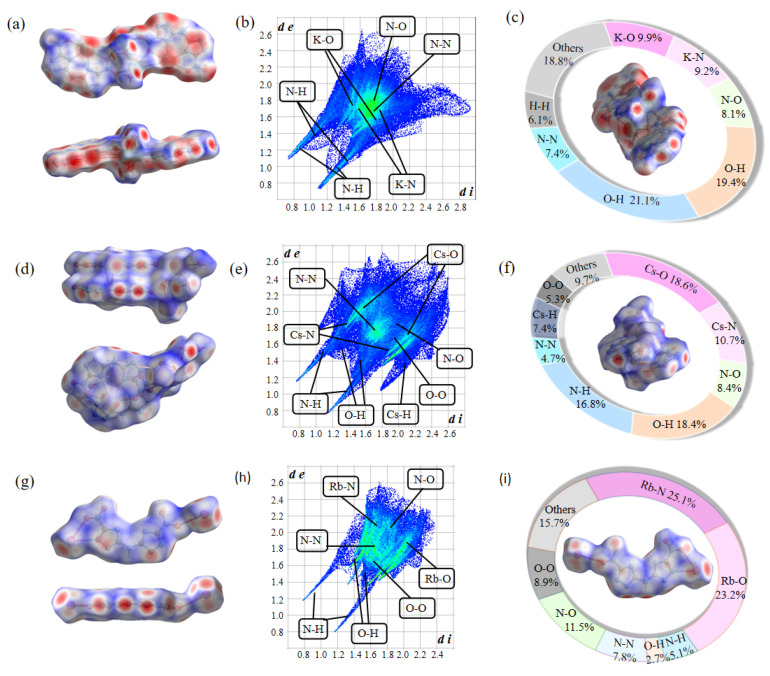
(**a**) Hirshfeld surface of compound **3**. (**b**) The 2D fingerprint plot of compound **3**. (**c**) The related individual atomic interaction proportion of compound **3**. (**d**) The Hirshfeld surface of compound **4**. (**e**) The 2D fingerprint plot of compound **4**. (**f**) The related individual atomic interaction proportion of compound **4**. (**g**) The Hirshfeld surface of compound **5**. (**h**) The 2D fingerprint plot of compound **5**. (**i**) The related individual atomic interaction proportion of compound **5**.

To gain a deeper understanding of the correlations between the physicochemical properties and intermolecular interactions of compounds **3**–**5**, Hirshfeld surface analysis, 2D fingerprint spectra, and individual atomic interaction proportions were conducted [53]. In compound **3**, the widely distributed red regions on the Hirshfeld surface (Figure 6a,c) represent strong coordination interactions between K–O and K–N atoms, as well as hydrogen bonding interactions. In contrast, blue and white spots indicate weaker π–π contacts between different layers of the molecule [54,55]. These close contacts are primarily attributed to nitrogen atoms on the 1,2,4-triazole ring and oxygen atoms from nitro groups or water molecules. Consequently, N–H and O–H hydrogen bonding interactions dominate the crystal structure, accounting for up to 40.5%. Coordination interactions between K–O and K–N atoms contribute an additional 19.1%. Additionally, N–N and N–O contacts, which are closely related to π–π interactions, account for 7.4% and 8.1%, respectively.

Compound **4** exhibits a similar pattern of weak non-covalent interactions to compound **3**. As shown in Figure 6d,f, hydrogen bonding and coordination bonds play a major role in its crystal structure. N–H and O–H hydrogen bonding interactions account for 35.2%, while K–O and K–N coordination interactions reach 29.3%. In contrast, compound **5** lacks water molecules, and coordination bonds are the primary driving force in its structure rather than hydrogen bonding (Figure 6g,i). Rb–N and Rb–O coordination bonds dominate the crystal structure of compound **5**, accounting for over 48.0%, while hydrogen bonding interactions contribute only 7.8%. Additionally, π–π stacking interactions mediated by N–N, O–O, and N–O contacts are present, contributing 7.8%, 8.9%, and 11.5%, respectively. This phenomenon is primarily attributed to the more parallel alignment of anionic planes in compound **5**.

The 2D fingerprint spectrum provides a visual representation of the distribution of weak interactions in these compounds. In all three compounds (**3**–**5**), two prominent spikes are observed, corresponding to high proportions of O–H and N–H hydrogen bonding (Figure 6b,e,h). The distribution of this hydrogen bonding is like some rich nitrogen E-MOFs, such as potassium 5-(hydrazine carbonyl)-3,4-dinitropyrazole [45]. The presence of these hydrogen bonds can effectively improve the stability of compounds and reduce their mechanical sensitivity to external stimuli. The high nitrogen and oxygen content in these compounds enhances molecular density while also reducing mechanical sensitivity. However, the abundance of coordination bonds and π–π interactions significantly contributes to the insensitivity of compounds **3**–**5** to external mechanical stimuli. The synergistic modulation of multiple weak interactions, including but not limited to hydrogen bonding, π–π stacking, and coordination bonding, represents a proven and versatile strategy for engineering E-MOFs with simultaneously enhanced thermal stability and reduced mechanical sensitivity.

## 3. Methods

The reagents and drugs used in this work were purchased from Aladdin Reagent Company (Shanghai, China). The synthetic route for compounds **2**–**5** is depicted in Figure S1. The starting material [56,57], Bis(3-nitro-1*H*-1,2,4-triazol-5-yl) amine (1), was synthesized based on our previous work. Utilizing compound **1** as the precursor, four E-MOFs were successfully obtained via a metathesis reaction [58,59]. Herein, the preparation method for compound **2** is described in detail. In a typical experiment, an ethanol solution of BNTA was first prepared. Sodium carbonate and water were then sequentially added to this solution. The reaction mixture was subsequently heated to 85 °C and maintained at this temperature for two hours. During the heating process, a substantial amount of precipitate formed. This precipitate was filtered, washed, and dried to yield a brown-yellow solid powder. These samples were characterized by ^1^H NMR, ^13^C NMR (400 AVANCE, Bruker, Biospin, AG, Ettlingen, Germany), IR (Spectrum II), single-crystal X-ray diffraction (D8 Venture, Bruker, AXS, Karlsruhe, Germany), and powder X-ray diffraction, etc. The details for all compounds (**2**–**5**) are provided in the Supplementary Materials.

## 4. Conclusions

In summary, a series of alkali metal-based energetic metal–organic frameworks (E-MOFs) were rapidly and conveniently synthesized using nitrogen-rich imino-bridged bis(1,2,4-triazole) as the ligand. All compounds (**2**–**5**) were thoroughly characterized. Structural studies of these E-MOFs revealed diverse coordination modes and three-dimensional architectures. These compounds feature not only abundant coordination bonds and hydrogen bonds but also significant π–π interactions, as evidenced by Hirshfeld surface analysis. These intermolecular interactions collectively contribute to their mechanical insensitivity. In terms of properties, compounds **2**–**5** exhibit high densities, ranging from 1.860 to 2.756 g·cm^−3^. Additionally, they possess high thermal decomposition temperatures and excellent mechanical stability against external stimuli. Notably, compound 3 stands out with a density of 1.941 g·cm^−3^, a high decomposition temperature of 285 °C, and low mechanical sensitivity (impact sensitivity >40 J; friction sensitivity >360 N). It also demonstrates remarkable detonation performance, with a detonation velocity of 8844 m·s^−1^ and a detonation pressure of 26.88 GPa, surpassing that of the traditional explosive RDX. These experimental and computational results clearly demonstrate that constructing E-MOFs using nitrogen-rich energetic molecules is an effective strategy to simultaneously enhance thermal stability and energy performance while reducing mechanical sensitivity to external stimuli. This approach paves the way for the development of next-generation energetic materials with optimized performance.

## Figures and Tables

**Table 1:** Physicochemical and energetic properties of compounds **2**–**5** in comparison with traditional explosives TNT, RDX, and HMX.

Compd.	*T_d_* ^a^ [°C]	*ρ* ^b^ [g·cm^−3^]	Δ*_f_H* ^c^ [kJ·mol^−1^]	*D* ^d^ [m·s^−1^]	*P* ^e^ [GPa]	*IS* ^f^ [J]	*FS* ^g^ [N]
**2**	283	1.860	−273.4	7116	17.51	>40	>360
**3**	285	1.941	−429.5	8844	26.88	>40	>360
**4**	287	2.756	−485.0	-	-	15	240
**5**	285	2.582/2.556 ^h^	−491.5	-	-	20	288
[Na(H_2_BTT) (H_2_O)_2_] _n_ [38]	336	1.706	-	8120	22.83	>40	>360
[Cs (ABTNA) H_2_O] _n_ [41]	225	2.413	974.05	6780	23.9	60	360
TNT [50]	295	1.65	−67.0	6881	19.5	15	353
RDX [51]	205	1.81	86.3	8750	34.2	7.5	120
HMX [52]	279	1.90	116.1	9144	41.5	7	112

^a^ temperature of decomposition; ^b^ density measured at room temperature; ^c^ calculated molar enthalpy of formation; ^d^ calculated detonation velocity; ^e^ calculated detonation pressure; ^f^ impact sensitivity; ^g^ friction sensitivity; ^h^ density at 298.15 K converted by formula *ρ*_(298.15K) =_ *ρ* − 0.188 × (298.15 − T)/1000.

## Data Availability

CCDC 2454221–2454223 contain the supplementary crystallographic data for this paper. These data can be obtained free of charge via www.ccdc.cam.ac.uk/data_request/cif (accessed on 27 April 2025), or by emailing data_request@ccdc.cam.ac.uk, or by contacting.

## Supplementary Materials

The following supporting information can be downloaded at: https://www.mdpi.com/article/10.3390/molecules30122478/s1, Experimental details, potential hazards, NMR spectra, mass spectra, IR spectra, crystallographic data, and theoretical calculations, etc. Refs. [60,61] are cited in Supplementary Materials.
