# Supplementary material for: Synthesis and Properties of Energetic MOFs Based on Bis(3-Nitro-1H-1,2,4-triazole-5-yl) Amine: Advancing High Thermal Stability and Low Sensitivity
Source: Molecules. 2025 Jun 6;30(12):2478. doi: 10.3390/molecules30122478 (PMC12195771; doi:10.3390/molecules30122478)
Supplement: Supplementary file 1 [file molecules-30-02478-s001.zip › molecules-3642537-supplementary.pdf]

Supporting Information

Synthesis and Properties of Energetic MOFs Based on Bis(3-Nitro-1H-1,2,4-triazole-5-yl) Amine: Advancing High Thermal Stability and Low Sensitivity

Shiluo Chen [1,2], Jinxin Wang [2], Yuteng Cao [2], Kangcai Wang [2], Haijun Yang [1,*], Tianlin Liu [2,*]

1. School of Materials and Chemistry, Southwest University of Science and Technology, Mianyang 621010, China; cslswust@163.com
2. Institute of Chemical Materials, China Academy of Engineering Physics, Mianyang 621900, China; wangjinxin20@gscaep.ac.cn (J.W.); caoyuteng18@gscaep.ac.cn (Y.C.); wangkangcai@caep.cn (K.W.)

* Correspondence: yanghaijun@swust.edu.cn (H.Y.); hollandtian@caep.cn (T.L.)

1. Synthetic Method 2
2. Single Crystal Data of Compound 3-5 4
3. Powder X-ray Diffraction of Compounds 3-5 7
4. Microscopic Image of Compound 3-5 8
5. Infrared Spectrum of Compound 1-5 9
6. Nuclear Magnetic Spectrum of Compound 1-4 9
7. Mass Spectrometry Spectrum of Compound 1 13
8. Theoretical Calculation 13
9. References 13

1. *Synthetic Method*

Caution: In the synthesis involved in this work, there may be the use of highly toxic, easily sublimated, or strongly alkaline chemical reagents. Relevant protective measures should be taken during use to avoid harm from drugs. In addition, both the synthesized precursor and target compound are energetic compounds, which exist certain safety hazards. The use of black metal spoons and external impacts should be avoided.

Bis (3-nitro-1*H*-1,2,4-triazole-5-yl) amine (1): The synthesis of compound **1** was obtained by the oxidative cleavage of C—N of the precursor compound, which was prepared according to the literature method using 1,3-diaminoguanidine hydrochloride as the raw material and undergoing two cyclization reactions under the action of cyanide bromide [S1,S2]. The detailed synthesis steps were as follows: 1,3-diaminoguanidine hydrochloride (20 mmol, 2.57 g) was added to a mixed solution of methanol (36 mL) and water (6 mL) at room temperature. Under stirring, cyanogen bromide (30 mmol, 3.28 g) was added to the above reaction solution. Then gradually raised the temperature to 85 ℃ and reacted for 4 hours. After the reaction was complete, the precipitate formed was collected by filtration. Disperse the collected solid (10 mmol, 1.95 g) in N,N'-dimethylfor- mamide (15 mL), and then sodium hydroxide (11 mmol, 440 mg) and cyanogen bromide (5.5 mmol, 602 mg) were sequentially added to the above dispersion under stirring. After addition, slowly increasing the reaction temperature to 85 ℃ and reacting for 4 hours. After reacting completely, the 5/6/5 tricyclic fused rings (TCS) precipitate formed was collected by filtration, washed and dried. TCS (1.89 mmol, 600 mg) was slowly dispersed in sulfuric acid (7.5 mL) in an ice bath environment, maintained for 30 minutes, and then dropped it into a sodium nitrite aqueous solution. After the dropwise addition was complete, continued stirring for additional 20 minutes and slowly raised the temperature to 40 ℃ for 2 hours. During this process, it could be observed that the reaction solution became clear, and then gradually solidified. The precipitate was collected by filtration, and the filter cake was dispersed in a small amount of deionized water. It was acidified with 20wt% sulfuric acid to a pH close to 3-4, forming a yellow precipitate. The precipitate was collected by filtration, ice water washing, and air drying, 290 mg (**1**, yield of 64%) of yellow solid was obtained. ^{1}H NMR (400 MHz, DMSO-d_6): δ (ppm) 11.60 ; ^{13}C NMR (100 MHz, DMSO-d_6): δ (ppm) 160.76, 150.98; IR (KBr pellet): 3482, 3281, 2722, 1627, 1567, 1521, 1416, 1308, 1149, 1090, 1051, 1012, 868, 848, 820, 719, 686, 553 cm^{-1}. m/z($C_{12}H_{12}N_7S$): calculation: 240.0435; found: 240.1468.

Bis (3-nitro-1*H*-1,2,4-triazole-5-yl) amine disodium salt (2): At room temperature, compound **1** (1 mmol, 241 mg) was dissolved in anhydrous ethanol (10 mL), and sodium carbonate (1.1 mmol, 117 mg) and a small amount of water were added while stirring. The solution gradually turned into a brick-red suspension. After adding, slowly raise the temperature to 85 ℃ and reacted for 2 hours. Filter and wash several times with a small amount of anhydrous ethanol. After air drying, obtain 281 mg (**2**, yield of 87%) of brownish red solid was obtained. ^{1}H NMR (400 MHz, DMSO-d_6): δ (ppm) 9.58 (s, 1H); ^{13}C NMR (100 MHz, DMSO- d_6): δ (ppm) 162.94, 160.33; IR (KBr pellet): 3241, 3081, 3002, 2836, 2669, 2597, 2413, 2131, 2009, 1858, 1814, 1613, 1530, 1490, 1431, 1362, 1315, 1285, 1241, 1082, 1039, 1007, 854, 817, 734, 650, 569, 539 cm^{-1}.

Bis (3-nitro-1*H*-1,2,4-triazole-5-yl) amine dipotassium salt (3): At room temperature, compound **1** (1 mmol, 241 mg) was dissolved in anhydrous methanol (10 mL), and potassium hydroxide (2.2 mmol, 124 mg) methanol solution was added dropwise while stirring. The solution quickly turned into a brownish red suspension, and after stirring at room temperature for 2 hours, it was filtered and washed several times with a small amount of anhydrous ethanol. After air drying, 294 mg (**3**, yield of 92%) of brownish red solid was obtained. ^{1}H NMR (400 MHz, DMSO-d_6): δ (ppm) 9.32 (s, 1H); ^{13}C NMR (100 MHz, DMSO- d_6): δ (ppm) 163.37, 160.42; IR

(KBr pellet): 3227, 3126, 3073, 3020, 2947, 2832, 2611, 2423, 2107, 1856, 1624, 1534, 1503, 1441, 1382, 1327, 1290, 1244, 1079, 1050, 1023, 858, 817, 739, 649, 550 cm^{-1}.

Bis (3-nitro-1*H*-1,2,4-triazole-5-yl) amine dicesium salt (4): At room temperature, compound **1** (1 mmol, 241 mg) was dissolved in anhydrous ethanol (10 mL), and cesium carbonate (1.1 mmol, 358 mg) and a small amount of water were added while stirring. The solution gradually turned into a brick-red suspension. After adding, slowly raise the temperature to 85 °C and reacted for 2 hours. Filter and wash several times with a small amount of anhydrous ethanol. After air drying, obtain 375 mg (**4**, yield of 74%) of brick red solid. ^{1}H NMR (400 MHz, DMSO-d_6): δ (ppm) 8.98 (s, 1H); ^{13}C NMR (100 MHz, DMSO- d_6): δ (ppm) 163.53, 160.50; IR (KBr pellet): 3251, 3069, 2998, 2835, 2595, 2410, 2131, 2094, 1875, 1815, 1619 1522, 1491, 1443, 1422, 1371, 1320, 1286, 1241, 1068, 1038, 1010, 847, 822, 733, 649, 543 cm^{-1}.

Bis (3-nitro-1*H*-1,2,4-triazole-5-yl) amine dirubidium salt (5): At room temperature, compound **1** (1 mmol, 241 mg) was dissolved in anhydrous ethanol (10 mL), and while stirring, rubidium carbonate (1.1 mmol, 254 mg) and a small amount of water were added. The solution gradually turned into a brick red suspension. After adding, slowly raise the temperature to 85 °C and react for 2 hours. Filter and wash multiple times with a small amount of anhydrous ethanol. After air drying, obtain 342 mg of brick red solid (**5**, yield of 83%). ^{1}H NMR (400 MHz, DMSO-d_6): δ (ppm) 8.62 (s, 1H); ^{13}C NMR (100 MHz, DMSO- d_6): δ (ppm) 163.37, 160.28; IR (KBr pellet): 3248, 3070, 3012, 2841, 2601, 2411, 2142, 2104, 1868, 1827, 1620, 1525, 1492, 1428, 1364, 1324, 1289, 1244, 1105, 1071, 1043, 1012, 851, 819, 738, 650, 615, 570, 545 cm^{-1}.

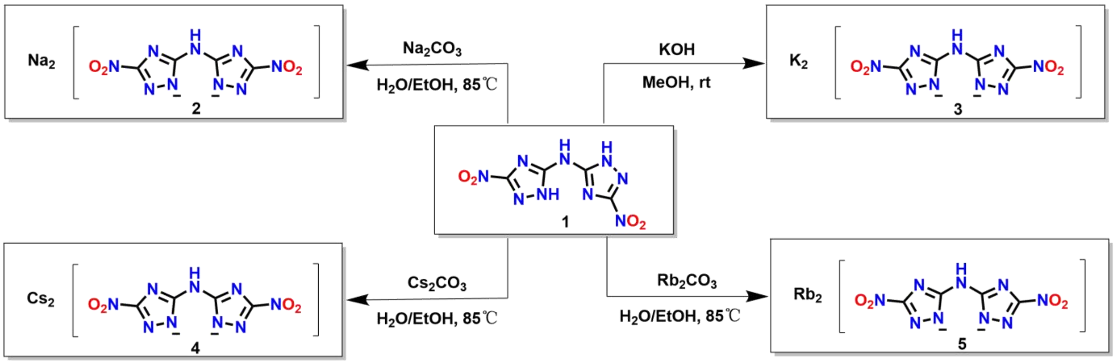

Figure S1. Synthetic route of compound 2-5

2. *Single Crystal Data of Compound 3-5*

Table S1. Crystal data and structure refinement for compound 3

	**Compound 3**
empirical formula	$C_4H_5N_9O_6K_2$
formula mass	353.37
temperature / K	295
Radiation	CuKα (λ = 1.54184)
crystal system	*triclinic*
space group	*P*-1
a / Å	9.7403(2)
b / Å	10.0212(2)
c / Å	12.5535(3)
α/（°）	98.134(2)
β/（°）	98.664(2)
γ/（°）	92.4562(19)
volume / $Å^3$	1196.59(5)
Z	2
*D*c/ $g·cm^{-3}$	1.941
F（000）	712
theta range for data collection/（°）	7.186 to 155.308
index ranges	$-12 \le h \le 12$, $-12 \le k \le 12$, $-15 \le l \le 15$
reflections collected	37949
independent reflections	5084 [R_{int} = 0.0510, R_{sigma} = 0.0231]
data / restraints / parameters	5084/5/407
goodness-of-fit on F^2	1.091
final *R* indices［I>2sigma（I）］	R_1 = 0.0333, wR_2 = 0.0893
*R*indices（all data）	R_1 = 0.0560, wR_2 = 0.1157
largest diff. peak and hole/$e·Å^{-3}$	0.56/-0.63

Table S2. Crystal data and structure refinement for compound 4

	Compound 4
Empirical formula	$C_8H_8N_{18}O_{11}Cs_4$
Formula weight	1063.96
Temperature/K	293(2)
Crystal system	monoclinic
Space group	$P2_1/c$
a/Å	7.2526(6)
b/Å	12.8737(10)
c/Å	27.8155(17)
α/°	90
β/°	90
γ/°	90
Volume/Å^3	2597.1(3)
Z	4
ρ_{calc}g/cm^3	2.721
μ/mm^{-1}	44.232
F(000)	1960
Crystal size/mm^3	0.24 × 0.12 × 0.1
Radiation	CuKα (λ = 1.54178)
2Θ range for data collection/°	7.566 to 156.098
Index ranges	$-9 \le h \le 8$, $-16 \le k \le 16$, $-22 \le l \le 35$
Reflections collected	12742
Independent reflections	4892 [R_{int} = 0.0594, R_{sigma} = 0.0715]
Data/restraints/parameters	4892/1/377
Goodness-of-fit on F^2	1.088
Final R indexes [I>=2σ (I)]	R_1 = 0.0557, wR_2 = 0.1573

Table S3. Crystal data and structure refinement for compound 5

	Compound 5
empirical formula	$C_4H_1N_9O_4Rb_2$
formula mass	410.08
temperature / K	150
Radiation	MoKα (λ = 0.71073)
crystal system	*monoclinic*
space group	$P2_1/n$
a / Å	7.2270(14)
b / Å	7.1174(13)
c / Å	20.724(4)
α/（°）	90
β/（°）	98.292(5)
γ/（°）	90
volume / $Å^3$	1054.9(3)
Z	4
*D*c/ g·cm^{-3}	2.582
F（000）	776
theta range for data collection/（°）	5.696 to 49.974
index ranges	$-8 \le h \le 7$, $-8 \le k \le 8$, $-23 \le l \le 24$
reflections collected	14470
independent reflections	1906 [R_{int} = 0.1015, R_{sigma} = 0.0788]
data / restraints / parameters	1906/120/173
goodness-of-fit on F^2	1.146
final *R* indices［*I*>2sigma（*I*）］	R_1 = 0.0794, wR_2 = 0.1636
*R*indices（all data）	R_1 = 0.1244, wR_2 = 0.1786
largest diff. peak and hole/e·$Å^{-3}$	2.41/-2.62

3. Powder X-ray Diffraction of Compounds 3-5

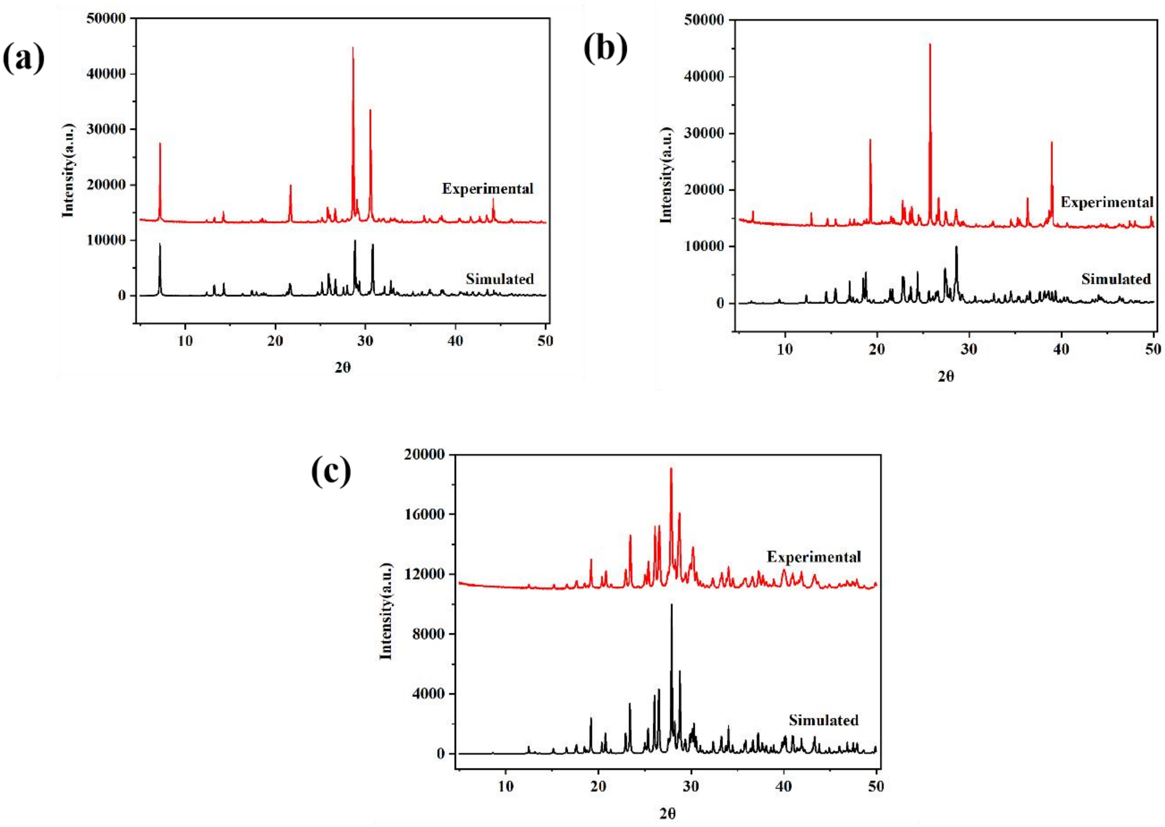

Figure S2.（a）PXRD of compound 3；（b）PXRD of compound 4；（c）PXRD of compound 5

4. Microscopic Image of Compound 3-5

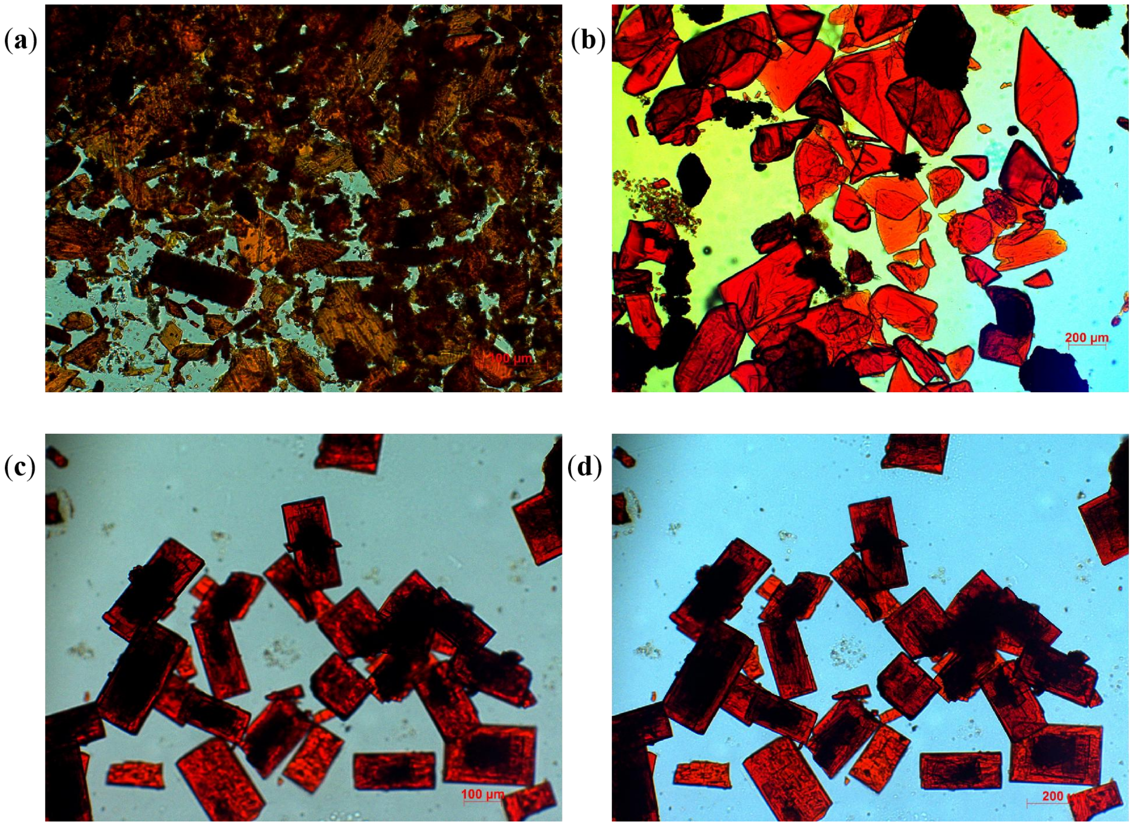

Figure S3.（a）Microscopic image of compound 3 at 100 um；（b）Microscopic image of compound 4 at 200 um；（c）Microscopic image of compound 5 at 100 um;（d）Microscopic image of compound 5 at 200 um.

5. Infrared Spectrum of Compound 1-5

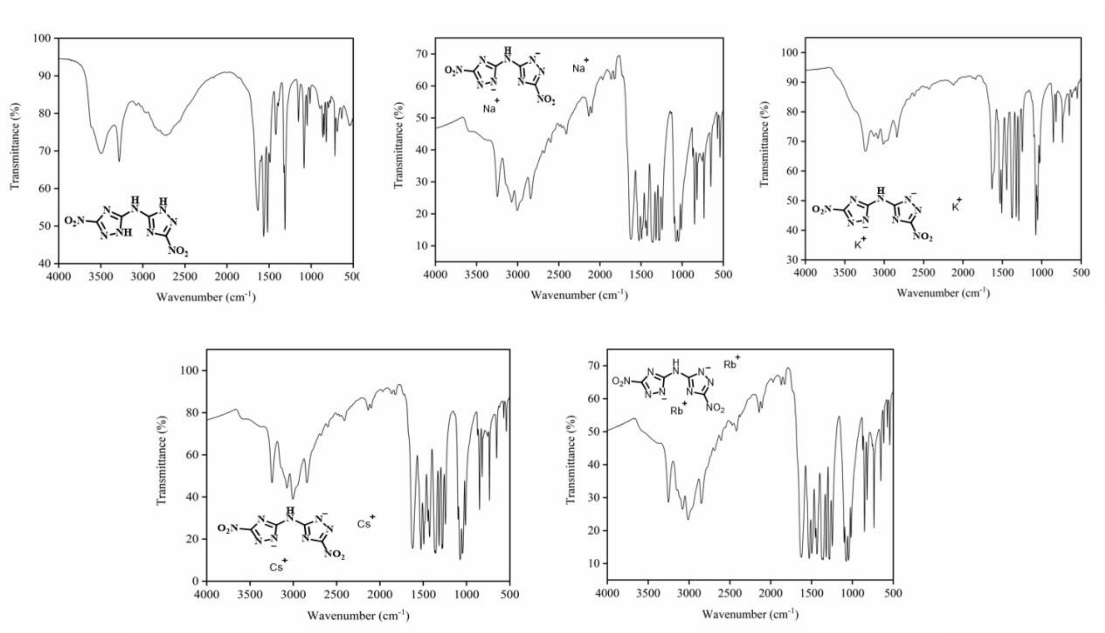

Figure S4. Infrared spectrum of compound 1-5

6. Nuclear Magnetic Spectrum of Compound 1-4

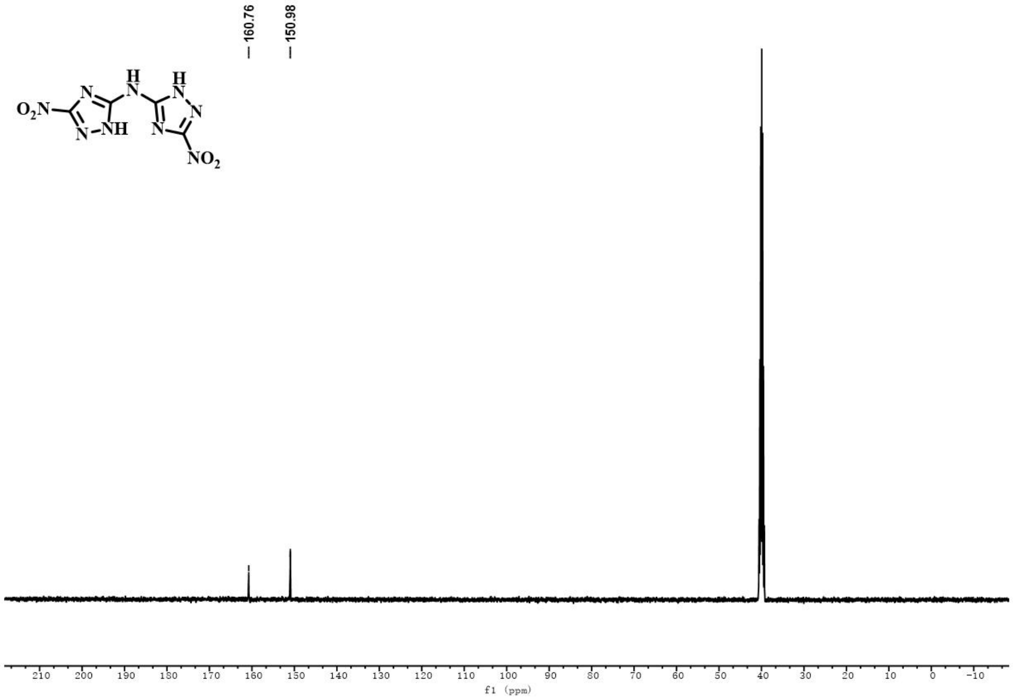

Figure S5. ^{13}C NMR of compound 1

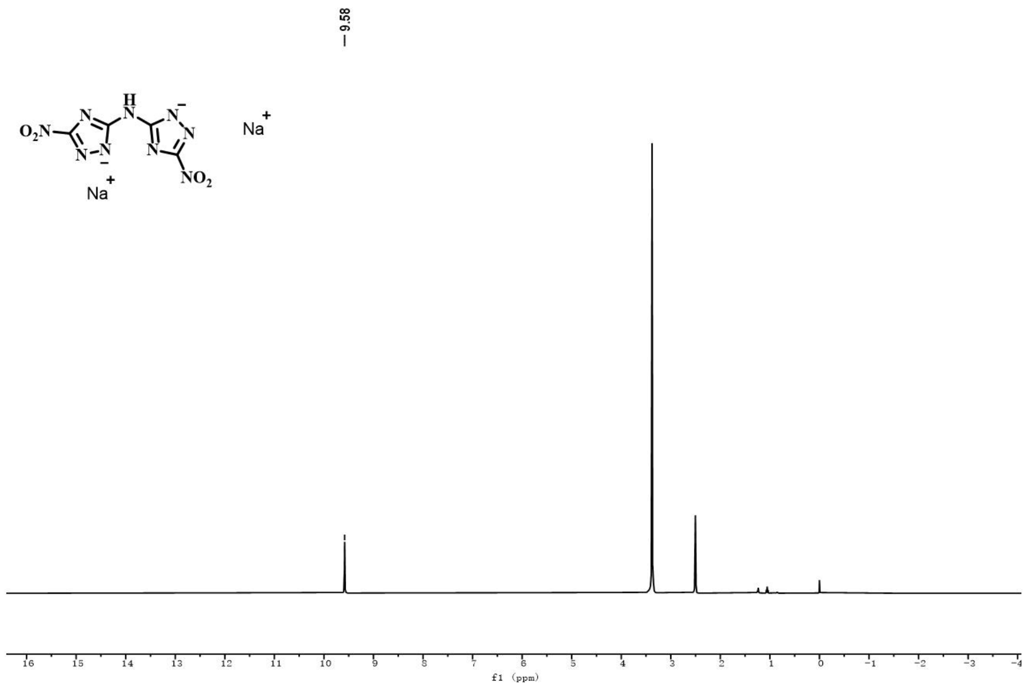

Figure S6. ^{1}H NMR of compound 2

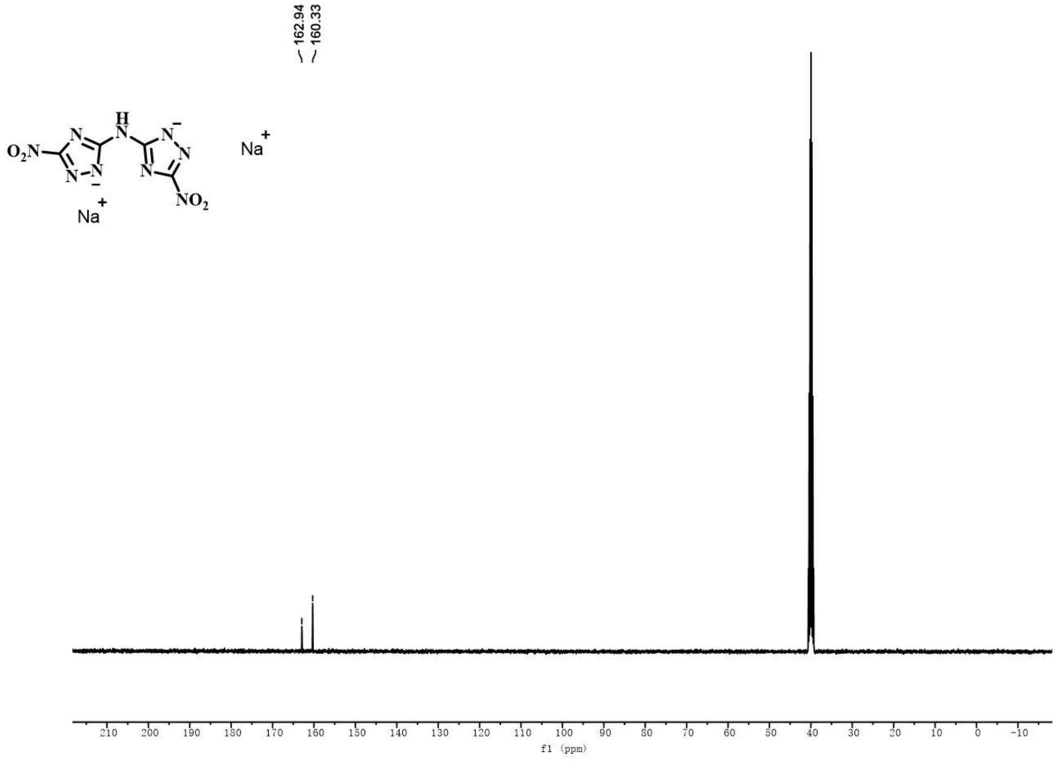

Figure S7. ^{13}C NMR of compound 2

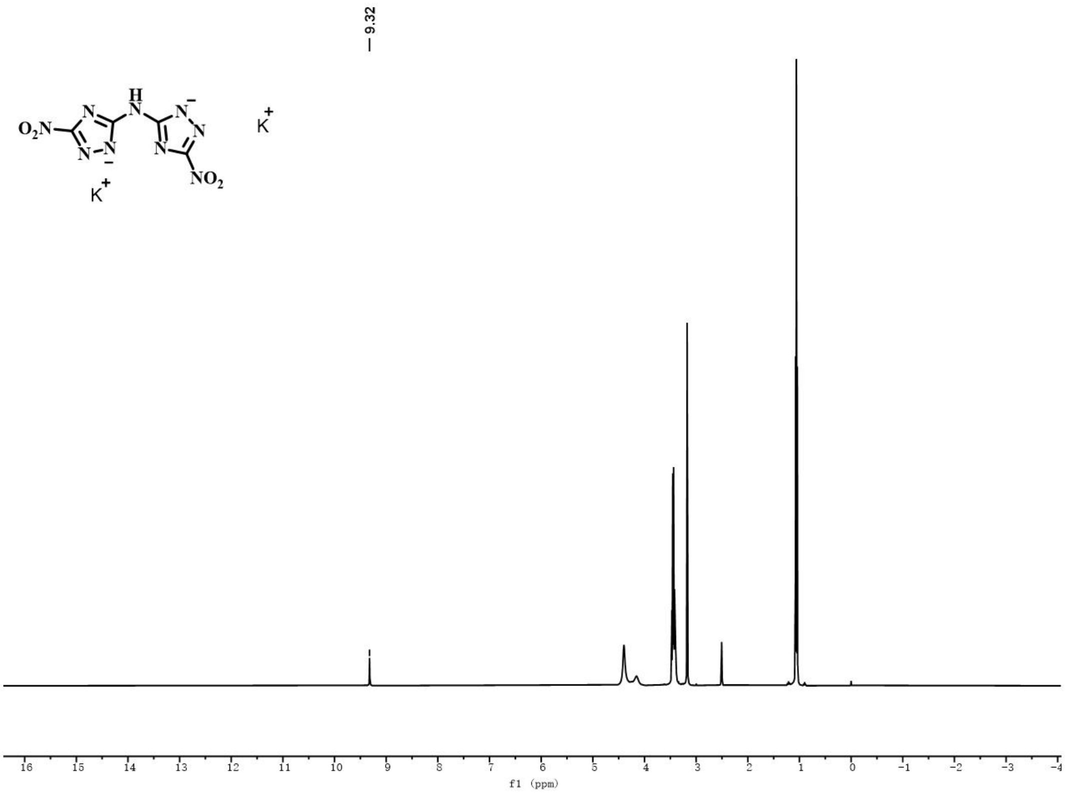

Figure S8. ^{1}H NMR of compound 3

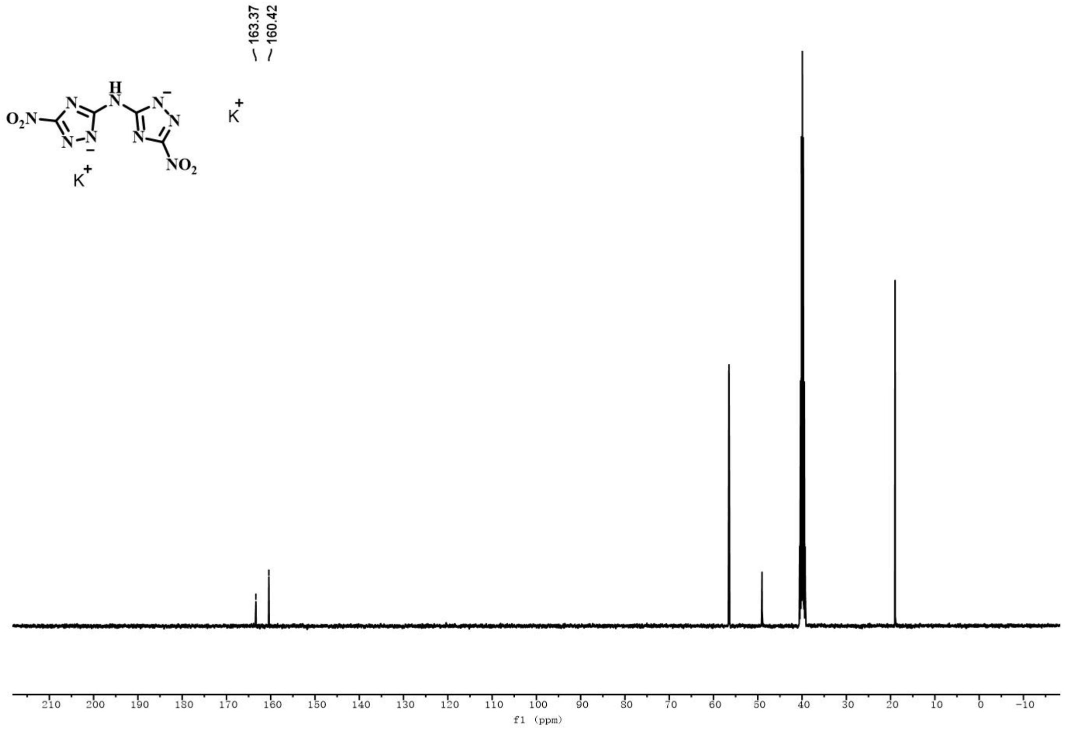

Figure S9. ^{13}C NMR of compound 3

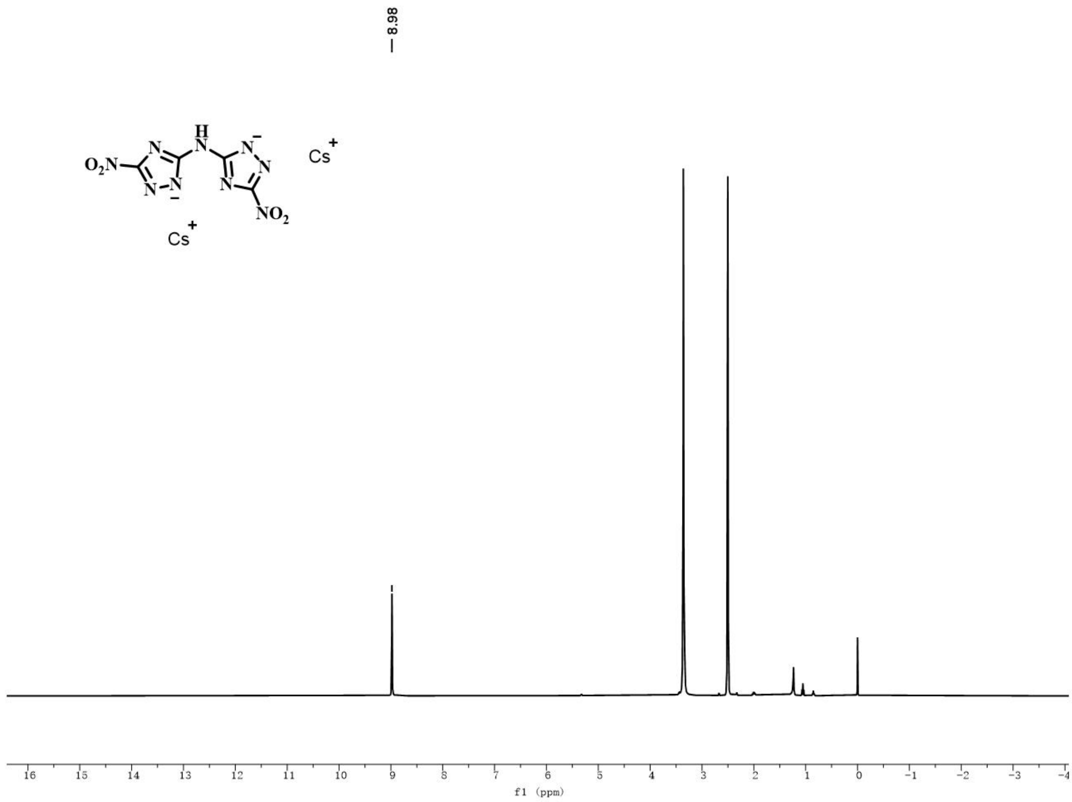

Figure S10. ^{1}H NMR of compound 4

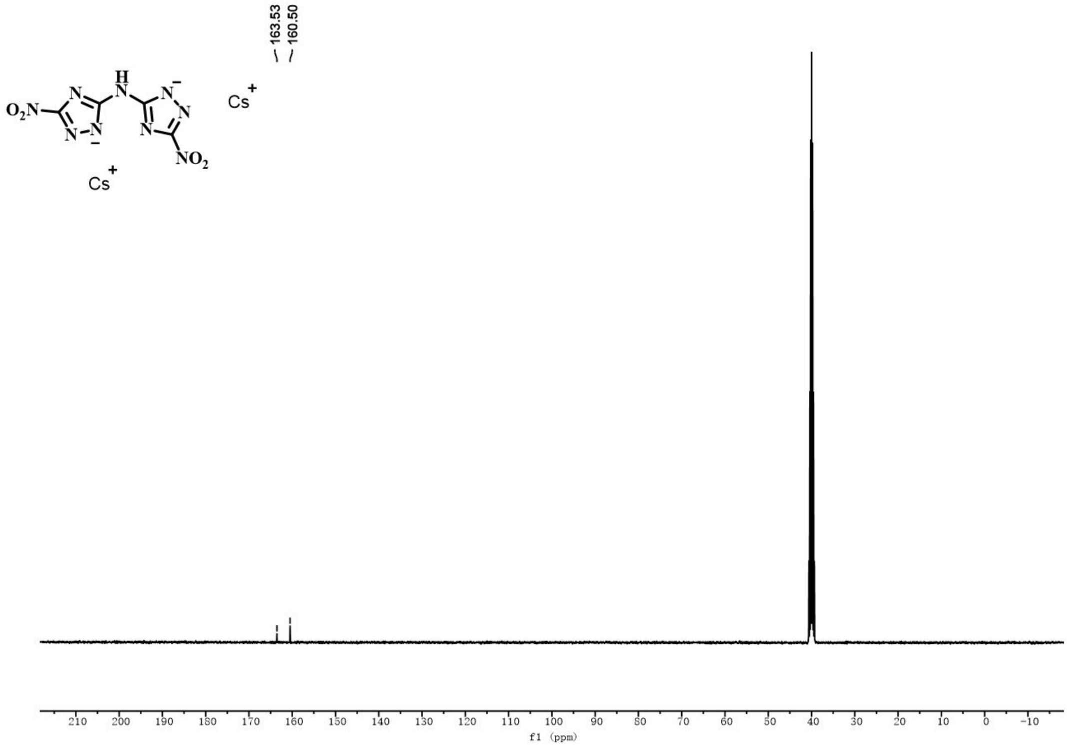

Figure S11. ^{13}C NMR of compound 4

7. Mass Spectrometry Spectrum of Compound 1

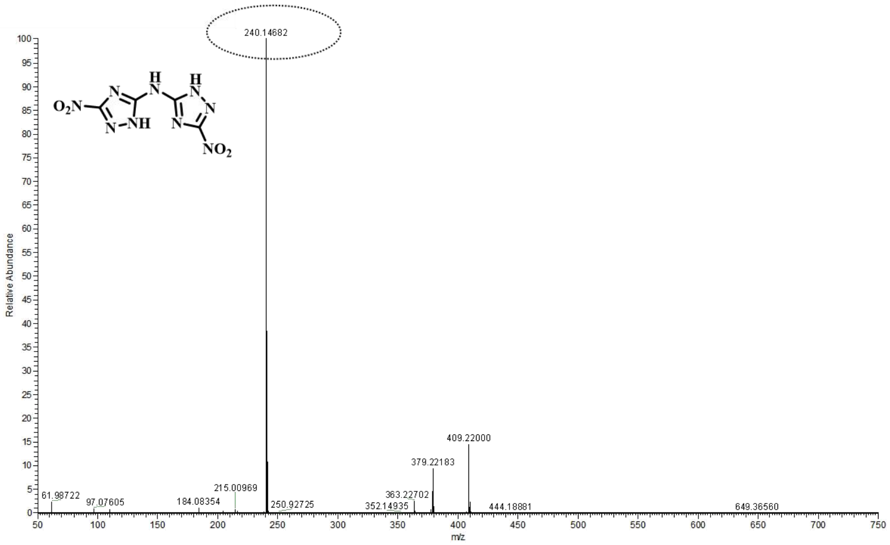

Figure S12. Mass spectrometry spectrum of compound 1

8. Theoretical Calculation

Theoretical calculations of heats of formation (HOF) were carried out by using the Gaussian 09 (RevisionD.01) suite of programs [S3]. For compounds 2-5, the solid-state heats of formation were calculated according to equation (1). In the equation (1), enthalpy of lattice (ΔH_L) was estimated by equation (2) and (3). In equation (2) and (3), "*p*", "*q*" are charge number for cation and anion; n_M, n_X, γ, and δ are constant decided by structure of ion; ρ_m and M_m are density molecular weight. More details about the parameters for equation (2) and (3) can be found in the published work [S4].

$$\Delta \mathrm{H_f}(ion, S) = m\Delta \mathrm{H_f}(cation, g) + n\Delta \mathrm{H_f}(anion, g) - \Delta \mathrm{H}_L \quad (1)$$

$$\Delta \mathrm{H}_L = U_{\mathrm{pot}} + \left[p\left({}^{n_M}\!/_2 - 2 \right) + q\left({}^{n_X}\!/_2 - 2 \right) \right] RT \quad (2)$$

$$U_{\mathrm{pot}} = \gamma\left(\rho_m / M_m\right)^{1/3} \quad (3)$$

9. References

S1. Zhou, Z.M.; Li, C.; Deng, C.L.; Wang, M. Structure Preparation Method and Performances of High Energy Heat Resistant Explosive CPTY[P]. Beijing: CN201711400638A, 2018–09–07.

S2. John, W.F.; Robert, D.C.; Richard, D.G. Facile Entry into the 3*H*,9*H*-Bis[1,2,4]Triazolo-[1,5-a:5′,1′-d][1,3,5]Triazinium (5/6/5 Tricyclic NNN) System[J]. *Tetrahedron Letters*. 2006, 47, 7707–7709.

S3. Frisch, M.J.; Trucks, G.; Schlegel, H.B.; Scuseria, G.E.; Gaussian 09, Revision D. 01; Gaussian Inc.: Wallingford C T, 2009.

S4. Jenkins, H.D.B.; Tudela, D.; Glasser, L. Lattice Potential Energy Estimation for Complex Ionic Salts from Density Measurements. *Inorg. Chem*. 2002, 41, 2364–2367
